# Group A Streptococcus Infections in Children: Epidemiological Insights Before and After the COVID-19 Pandemic

**DOI:** 10.3390/pathogens13111007

**Published:** 2024-11-15

**Authors:** Eleni Karapati, Andreas G. Tsantes, Zoi Iliodromiti, Theodora Boutsikou, Styliani Paliatsiou, Aglaia Domouchtsidou, Petros Ioannou, Vasileios Petrakis, Nicoletta Iacovidou, Rozeta Sokou

**Affiliations:** 1 Neonatal Department, Aretaieio Hospital, School of Medicine, National and Kapodistrian University of Athens, 11528 Athens, Greece; helenak5@hotmail.com (E.K.); ziliodromiti@yahoo.gr (Z.I.); theobtsk@gmail.com (T.B.); stpaliatsiou@yahoo.gr (S.P.); niciac58@gmail.com (N.I.); 2 Microbiology Department, “Saint Savvas” Oncology Hospital, 11522 Athens, Greece; ldomouchtsidou@gmail.com; 3 School of Medicine, University of Crete, 71003 Heraklion, Greece; p.ioannou@uoc.gr; 4 Department of Infectious Diseases, HIV Unit, 2nd University Department of Internal Medicine, University General Hospital of Alexandroupolis, Democritus University of Thrace, 68131 Alexandroupolis, Greece; vasilispetrakis1994@gmail.com; 5 Neonatal Intensive Care Unit, General Hospital of Nikaia “Agios Panteleimon”, 18454 Piraeus, Greece

**Keywords:** streptococcus group A, GAS, COVID-19, SARS-CoV-2, children, infection

## Abstract

Group A streptococcus (GAS) is the cause of both mild and invasive infections in humans with a high morbidity and mortality rate. The transmission of disease usually occurs via droplets, so the implementation of infection mitigation strategies (IMS) during the COVID-19 pandemic altered the incidence of GAS infection. This review aims to provide an overview of the influence of the COVID-19 pandemic on the incidence of GAS infection in children (invasive or non-invasive). A surge in the incidence of invasive GAS infection was noted in December 2022 after the reversal of IMS. A global uprise in GAS infection (invasive and non-invasive) was noted, especially concerning the pediatric population. Children younger than 5 years old were mostly affected, with complicated pneumonia being the leading clinical manifestation, causing many deaths worldwide. *Emm1*, specifically M1UK, was recognized as the dominant lineage in Europe and correlated with invasive disease. Healthcare professionals need to be alert about the severity of GAS-related infections, leading to early identification and treatment.

## 1. Introduction

Group A streptococcus (GAS), otherwise referred to as streptococcus pyogenes, is a Gram-positive coccus, causing a variety of infection spectra in humans, ranging from mild (pharyngitis, scarlet fever, tonsillitis, impetigo) to severe, invasive, and possibly life-threatening [1,2]. It is the leading cause of bacterial pharyngitis in children and adolescents. Invasive GAS infection (iGAS) is established with the isolation of GAS in a normally sterile site (such as blood, joint fluid, deep tissue, pleural fluid, CSF, etc.) or a usually nonsterile site in a patient with toxic shock syndrome [3]. iGAS infection can present with clinically different manifestations, which include pneumonia, cellulitis, osteomyelitis, arthritis, bacteremia without focus, necrotizing fasciitis, streptococcal toxic shock syndrome (STSS), meningitis, and endocarditis [4]. iGAS infection affects children and adults with or without comorbidities, although viral co-infection, immunodeficiency, skin disruption, diabetes, cancer, alcohol use, and extremity of age are identified as risk factors and are accompanied by a high mortality rate of up to 20% [5,6]. Immune-mediated manifestations caused by GAS, such as post-streptococcal glomerulonephritis, rheumatic heart disease, and rheumatoid fever, should also be highlighted as they portray a great health burden, especially in developing countries [7].

The highly invasive nature of GAS is attributed to many factors. M protein, a surface antigen of leading virulence, is encoded by the *emm* gene and has a crucial role in the pathogenesis of invasion and phagocytosis [8]. *Emm* sequence typing, mainly via the 5′ end of the M protein (*emm*) gene, is widely used in several epidemiological studies in Europe and the USA to observe changes in the geographical and temporal alteration of GAS infection, to assess invasive or non-invasive GAS infection rates, and to correlate *emm* typing with the occurrence of invasive disease [9,10]. More than 200 *emm* types have been described, with a great variation in regard to geographic location and season [11]. Most researchers concur that *emm1* and *emm3* are related to invasive disease. In addition, GAS can create pyrogenic exotoxins with superantigen capabilities, with streptococcal pyrogenic exotoxin A (SpeA) being the most studied. Finally, a plethora of extracellular products, such as DNases, hyaluronidases, peptidases, and streptokinases, are responsible for tissue and immune invasion [12].

GAS is a member of the microflora, colonizing the skin and the throat, with healthy carriers consisting of a natural reservoir of infection, occurring typically in winter and spring in temperate climates. GAS is usually transmitted via droplets by face-to-face contact or contaminated surfaces and rarely by wound pus or contaminated food products [13]. Therefore, infection mitigation strategies (IMS), such as those implemented in the COVID-19 pandemic, could influence the incidence of GAS infection as they limit droplet transition. Indeed, social distancing, universal mask implementation, restriction of public activities and school, intensification of hand hygiene, and surface cleaning led to a decrease in many pediatric infections such as gastroenteritis, common cold, pharyngitis, otitis, etc. [13].

At the end of the COVID-19 pandemic, when IMS were reversed, a rise in GAS infection was noted. On 12 December 2022, the World Health Organization (WHO) stated an increase in iGAS infection in several European countries, especially in the United Kingdom, France, Spain, Sweden, and Denmark, with the United Kingdom reporting a high rate of iGAS infection in children accompanied by several deaths in a brief timeframe [2,14,15]. A similar trend was noted by the Centers for Disease Control and Prevention (CDC) in February 2023 regarding iGAS infection in the USA [16,17].

The purpose of this review is to provide an overview of the influence of the COVID-19 pandemic on the incidence of GAS infection in children (invasive or non-invasive).

An electronic search of existing literature was conducted in the databases Pubmed and Scopus from September 2024 to October 2024. The search terms included “Streptococcus group A”, “COVID-19”, “children”, and “GAS”. Only studies published in English were included, and no geographical or time limitations were imposed. In total, a number of 6234 papers were retrieved (6121 in Scopus and 113 in Pubmed). After a careful screening of their titles and abstracts, 97 studies were considered eligible, and following the elimination of duplicate files, 67 studies were selected for a thorough full-text review. Following a thorough examination of the full text, 44 studies were finally included.

## 2. Epidemiological Data Regarding GAS Infection Before—During—After the Pandemic

Epidemiological data reflecting the influence of the COVID-19 pandemic on GAS infection are reported in many countries around the world. Details on the incidence of GAS infection before and after the COVID-19 pandemic are depicted in Table 1.

### 2.1. Epidemiological Data in Europe

The COVID-19 pandemic led to a decrease in GAS-related bloodstream infections, as reported by Amarsy et al. in Paris, France, in 2020 [18]. In a prospective study, Cohen et al. [19] additionally noted a decrease in non-invasive GAS infection in France in 2020, followed by a rise in the cases after March 2022 with an increase in a rate of +23.8% per month, as monitored via rapid antigen strep tests, mainly manifesting as tonsillopharyngitis. Ceano-Vivas et al. [4] reported a drop in GAS infections at ED visits during the pandemic years of 2020 and 2021 and a rise in the cases in 2022 when compared to the epidemiological data of the years 2018 and 2019. Still, the rise in GAS incidences was not statistically significant. Most cases in 2022 (97.3%) were mild cases of scarlet fever and streptococcal pharyngitis, while iGAS cases represented only 2.7%, of which the most common infection was pneumonia (25%). A resembling observation was made by Calvo et al. [20] when observing the GAS-related ER visits in Spain during 2018–2023. GAS-related ER visits in the first semester of 2023 were doubled when compared to 2022 and 2019. A similar rate was also noted regarding iGAS infection in the same time period. iGAS infection constituted a high percentage of pneumonia cases, having increased severity, while viral coinfection was common. Epidemiological data from Germany, as reported by Goretzki et al. [21], noted an outbreak of iGAS infection in the last trimester of 2022 in the pediatric population, with a median age of 4 years old, mainly presented as pneumonia, followed by STSS, with a high viral co-infection rate of 46% and a high ICU admission rate of 59% and death rate, when compared to statistics of pre-pandemic years. A similar observation is noted by Tomidis Chatzimanouil et al. [22] in a retrospective cohort study also conducted in Germany. They detected a rise of the iGAS infection rate in the pediatric population by 1200% in the first half of 2023 when compared to older data and a high occurrence of sepsis, STSS, and complicated pneumonia. Nevertheless, post-pandemic data report a shorter hospital stay and duration of antibiotic administration. Meanwhile, Singer et al. [23] provided information in regard to the incidences of bacterial infection in Germany in the years 2022–2023. They observed an uprise in iGAS infection in the first quarter of 2023 by 142% when compared to data from 2017 to 2019, with children younger than 5 years of age being the most affected. This observation coincided with a surge in viral co-infection in the same timeframe—an unusually high wave of Influenza A and RSV, followed by an equally strong wave of Influenza B. In the UK, a similar trend was noted by Iro et al. [24], with a drop in hospital admissions due to GAS from April 2020 to Spring 2022, when admission rates began to reach pre-pandemic levels. A shift in the seasonal peak was also underlined, with a peak in admission noted in December 2022, higher than every other month in the last 20 years. Three studies conducted in the UK report data about GAS pneumonia cases in children in the years 2022–2023. Wrenn et al. [25] acknowledged an increase in low respiratory tract infection in children in the UK in a case series conducted from October to December 2022. In total, 147 cases were registered, with a median age of 4 years of age, and 48% had a viral coinfection (mainly RSV or hMPV). A high death rate was underlined (25% of the cases), occurring after a sudden and rapid deterioration, mainly in the community. Another study by Lees et al. [26] included data about pediatric patients with GAS pneumonia with parapneumonic effusion in 2022–2023, with a median age of 4 years old. A total of 66% of the patients had a viral coinfection, mainly human metapneumovirus, varicella zoster, and influenza, and the majority required primary surgical management (92.4% of the cases), while 18.9% of the cases were complicated with STSS. The PICU admission rate reached 64.3%. A total of 16.8% of the patients had already received oral antibiotics before hospitalization. In a national study in the UK, Holdstock et al. [27] identified GAS-related pleural empyema cases in children. While until August 2022, Streptococcus Pneumoniae was the main pathogen, from September 2022, an uprise in the cases of GAS-related pneumonia was noted. A total of 60% of the cases reported a viral co-infection, mainly human metapneumovirus, respiratory syncytial virus, Influenza A, varicella-zoster, and rhinovirus. Half of the children admitted to the PICU required mechanical ventilation. Similar data are also reported in Poland by Grochowska et al. [28]. They conducted a retrospective analysis of 91 pediatric patients hospitalized for complicated community-acquired pneumonia (CAP) in Warsaw, Poland. GAS was featured as the main pathogen responsible for CAP, constituting 24.2% of the cases. A total of 68.2% of those patients presented with scarlet fever, and 27.3% reported viral coinfection that either preceded or coexisted with pneumonia. In total, they reported longer hospitalization, a higher occurrence of chest insertion, and higher procalcitonin levels in CAP by GAP than caused by other bacteria. Interestingly enough, a study conducted in 2016 in the same hospital revealed a lower percentage of GAS in complicated pneumonia (4.7–5.8% of the study group) [29]. Likewise, Peetermans et al. [5] reported a surge in pediatric CAP cases caused by GAS in Belgium in 2022–2023, complicated with empyema in 83% of the cases. They also reported a high viral coinfection rate. In Italy, Massese et al. [6], in a retrospective study of 1839 children, noted a decline of GAS-positive pharyngeal cultures during the COVID-19 pandemic and a subsequent surge during 2023. Notably, the GAS infection epidemiology changed during 2023, with cases showing no seasonal pattern, affecting mainly children between 3 and 5 years of age. Similarly, data are also described by Cinicola et al. [30], examining the occurrence of GAS infection in children visiting the family doctor in Italy during the first five months of 2023. A total of 20.3% of children reported GAS infection, most commonly diagnosed as pharyngotonsillitis treated with antibiotics. In Switzerland, Schöbi [31] et al. revealed data on GAS clinical outcome and management in the period 2013–2023. Although a surge in iGAS infection rate was noted in 2022–2023 at the same period of the Influenza A/B outbreak, mainly presented as pleural empyema and soft tissue infection, this did not lead to a longer hospital stay, a need for ICU admission, or septic shock. Data on invasive GAS infection are also presented in a case series by Coşkun et al. [32] in Turkey, describing the severity, management, and clinical outcome of three cases with STSS in the PICU. In a recent study published in 2024, Valcarcel Salamanca et al. [33] described a rise in iGAS infection in children during the first half of 2023 in Norway, mainly in children younger than 10 years old, that peaked in January 2024 with a threefold increase. Clinical presentation included sepsis, pleuritis, and empyema. A similar surge was also noted by the same study as to strep throat, with a peak in December 2023. In the Netherlands, Van Kempen et al. [34] surveyed invasive GAS infection in the pediatric population in 2022. A rise in iGAS infection occurred in the first semester of 2022 in children younger than 5 years of age, mainly presented as pneumonia complicated with empyema, while cases of necrotizing fasciitis were diminished. A total of 18% of the cases reported influenza infection in the previous timeframe. De Gier et al. [35] also noted an uprise in iGAS pediatric cases in the Netherlands in 2022, mainly in children younger than 5 years old.

### 2.2. Epidemiological Data in Australia

Abo et al. [36] reported an increase in iGAS cases in Australia in the last semester of 2022, affecting mainly children 5 years old. This surge came after a decline in cases during the pandemic. The fatality rate reached 1%, with pneumonia with empyema and bacteremia without the focus being the most commonly acquired manifestations in 2022, while during the pandemic years, soft tissue infections were more common. In regards to pneumonia, 32% of the cases were severe, and 56% of them required mechanical ventilation. Viral coinfection was associated with a severe course of pneumonia, with the main representers being rhinovirus or rhinovirus/enterovirus, human metapneumovirus, and adenovirus.

### 2.3. Epidemiological Data in the USA

A decrease in iGAS infection in the USA was noted by McNeil et al. [37] in 2020, especially regarding the incidence of deep abscesses. Boyanton et al. [13] also confirmed that IMS strategies led to a reduction in GAS pharyngitis by 81.6% during the pandemic years in the USA. Data from the USA in regards to GAS infection after the implementations of IMS strategies, mainly from Colorado and Minnesota, by Barnes et al. [38], underlined an uprise of iGAS infection in children in the last trimester of 2022, which followed the unusual decline of cases during 2020. This uprise occurred without the usual seasonal pattern, coincided with an increase in viral infection rates like RSV and Influenza, and affected mainly young children, with a median age of 3 years old. No change in the length of hospital stay, and PICU admission was noted. A total of 61.8% of the cases reported a prior viral infection. In another study carried out in the USA by Engstrom et al. [39], a decrease in the rates of RSV and Influenza during 2020 and the subsequent rise of their rate in 2022 is consistent with the pattern of the rate of iGAS infection during these years. A prospective study by Ho et al. [40], which took place between October 2022 and April 2023 in Colorado, assessed invasive GAS cases in children. A total of 60% of the patients reported viral coinfection, and 39% demanded PICU admission. Soft tissue disease, mainly occurring in the head, eyes, ears, nose, throat (HEENT), and musculoskeletal system, followed by pneumonia, was the most common manifestation. Pneumonia cases appeared with an extensive rise in 2022 when compared to pre-pandemic years. Nack et al. [41] studied iGAS cases in infants younger than 1 year of age from 2012 to 2022 in the USA. The most common clinical manifestation was pneumonia with or without empyema (21.8%), with most of them occurring during the last quarter of 2022, with a PICU admission rate of 59.4%.

**Table 1:** Studies on the incidence of GAS infection before and after the COVID-19 pandemic.

First Author, Publication Year	Country	Study Type	Time Period	Study Design	Study Population	Results
Grochowska et al. [28], 2024	Poland	cohort	2022–2023	retrospective	91 children < 18 years old	GAS was the dominant cause, constituting 24.2% (22/91; 95% CI 15.8–34.3%) of complicated community-acquired pneumonia
Ceano-Vivas et al. [4], 2024	Spain	cohort	2018–2022	retrospective	1739 children < 18 years old	GAS infections decreased during the COVID-19 pandemic. Mild and severe GAS cases increased considerably in 2022 but did not reach similar levels to those detected in other countries
Calvo et al. [20], 2024	Spain	observational	2018–2023	retrospective	N/A	Increase in GAS and iGAS infection in the first trimester of 2023 in Spain
Wren et al. [25], 2023	UK	case series	2022	retrospective	147 children < 15 years old	Unusual rise in iGAS LRTIs in children in late 2022 in the UK
Iro et al. [24], 2023	UK	observational	2000–2022	retrospective	N/A	Admission rates due to GAS infection continued to rise throughout the rest of the year 2022, reaching a record of 432 per 100,000 population in December 2022
Lees et al. [26], 2024	UK	case series	2022–2023	retrospective	185 children < 16 years old	Epidemiology of pediatric group A Streptococcus pneumoniae with parapneumonic effusion in the UK in the post-COVID-19 era
Holdstock et al. [27], 2023	UK	case series	2022	retrospective	16 children < 16 years old	A rise in pleural empyema associated with group A streptococcus (GAS) was noted from September to December 2022
Guy et al. [42], 2023	UK	observational	2022	retrospective	772 patients (adults and children)	Increases in invasive group A streptococcal (iGAS) infection and associated deaths, particularly in children, above seasonally expected levels are being seen this season
Nygaard et al. [43], 2024	Denmark	cohort	2016–2017, 2021–2023	retrospective	174 children < 18 years old	In Denmark, the incidence of pediatric iGAS increased in 2022–23 compared with the three preCOVID-19 seasons of 2016–17, 2017–18, and 2018–19, but the course of iGAS disease in 2022–23 was not more severe than in previous seasons
Cohen et al. [19], 2023	France	cohort	2018–2022	retrospective	11,701 children < 15 years old	In France, in 2020, the incidence of GAS diseases decreased by 84.5% (*p* < 0.001), with no significant trend until March 2022, when the incidence significantly increased (+23.8% per month, *p* < 0.001)
Amarsy et al. [18], 2023	France	observational	2018–2020	retrospective	N/A	Bloodstream infections (BSIs) due to Streptococcus pyogenes decreased during the two COVID-19 lockdown periods of 2020.
Massese et al. [6], 2024	Italy	observational	2018–2023	retrospective	1839 children < 18 years of age	The GAS positivity rate decreased from 13% in the pre-pandemic period (data from 2019) to 2% in 2022; eventually, the rate increased again to 13% in 2023
Cinicola et al. [30], 2024	Italy	observational	2023	retrospective	3580 children < 16 years old	The incidence of GAS infection reaches 20.3% in the post-COVID-19 era
Mangioni et al. [16], 2024	Italy	cohort	2022–2023	retrospective	179 patients (19 adults and 9 children)	Increased number of GAS infections in two university hospitals in Milan, Italy, in the last quarter of 2022 and the first quarter of 2023
Peetermans et al. [5], 2024	Belgium	cohort	2022–2023	retrospective	86 patients (56 adults and 30 children)	A high number of admissions to Belgian tertiary critical care units of patients with severe invasive *S. pyogenes* infections associated with the introduction of the M1UK lineage
Goretzki et al. [21], 2024	Germany	cohort	2022	retrospective	153 children	The unprecedented peak of bacterial infections and deaths in late 2022 and early 2023 was caused mainly by *S. pyogenes* and *S. pneumoniae*
Tomidis Chatzimanouil et al. [22], 2024	Germany	cohort	2015–2023	retrospective	178 patients (adults and children)	Children were at higher risk for iGAS infections post-pandemically, but it was not accompanied by increased iGAS-associated morbidity and mortality.
Singer et al. [23], 2024	Germany	observational	2017–2019, 2022–2023	retrospective	4885 invasive isolates	The number of invasive *S. pyogenes* isolates rose by 142% compared to pre-pandemic seasonal peak values
Schöbi et al. [31], 2024	Switzerland	observational	2013–2023	retrospective	284 children < 16 years old	No evidence supporting the hypothesis that the 2022–2023 GAS outbreak was associated with a change in preadmission management possibly induced by the new recommendation for GAS pharyngitis
Coşkun et al. [32], 2023	Turkey	case series	2023	retrospective	3 children	Three patients with STSS who were followed in the Pediatric Intensive Care Unit with *S. pyogenes* growth in blood and pleural fluid cultures in the last 2 weeks are presented.
Valcarcel Salamanca et al. [33], 2024	Norway	cohort	2015–2024	retrospective	2129 patients	During the first half of 2023, the number of invasive group A streptococcus (iGAS) notifications increased in Norway, followed by a new surge in December 2023 and peaking between January and February 2024
van Kempen et al. [34], 2022	Netherlands	observational	2018–2019, 2021–2022	retrospective	117 children < 18 years old	Pediatric iGAS case numbers were 2-fold higher between July 2021 and June 2022 versus pre-COVID-19 in the Netherlands.
de Gier et al. [35], 2023	Netherlands	observational	2022	retrospective	42 children 0–5 years old	In 2022, a sevenfold increase in the number of notifiable invasive *Streptococcus pyogenes* (iGAS) infections among children aged 0–5 years was observed in the Netherlands compared with pre-COVID-19 pandemic years
Barnes et al. [38], 2023	USA	observational	2016–2019, 2020–2021, 2022	retrospective	49 children < 18 years old	During fall 2022, a resurgence of invasive group A streptococcus (iGAS) infection in children and adolescents was observed in the USA
Boyanton Jr et al. [13], 2023	USA	case series	2018–2019, 2020–2021	retrospective	14,675 patients < 18 years old	After the implementation of infection mitigation strategies, the incidence of GAS-P dropped by 81.6%
McNeil et al. [37], 2021	USA	observational	2017–2020	prospective	269 children < 18 years old	With regards to IGAS, an increase in incidence was noted from 2017 to 2019, which was followed by a decline in 2020
Engstrom et al. [39], 2023	USA	observational	2018–2022	retrospective	210 children < 18 years old	Rates of iGAS infection decreased by 46% from 2019 to 2020. In 2022, a surge above pre-pandemic rates of iGAS infections was noted.
Ho et al. [40], 2023	USA	observational	2022–2023	prospective	96 patients < 21 years old	Outbreak case numbers of iGAS were almost triple the pre-pandemic baseline
Nack et al. [41], 2024	USA	observational	2012–2022	retrospective	32 infants < 1 year old	25% of iGAS cases in infants < 12 months of age occurred in the final quarter of 2022
Golden et al. [44], 2024	Canada	Observational	2021–2022	Retrospective	4809 patients	A rise in regards to iGAS infection at the end of 2022 is noted, especially in children < 15 years old
Abo et al. [36], 2023	Australia	observational	2018–2022	retrospective	280 children < 18 years of age	Australia experienced an increase in the incidence of iGAS among children and young people in 2022 compared to pandemic years 2020–2021, similar to northern hemisphere observations

## 3. Antibiotic Resistance

Antibiotic resistance is a cause of great concern. Different strains that emerge in different countries possess a variety of resistance; for example, GAS in China appears with a frequent resistance to macrolides and clindamycin [45]. Still, there is no indication of resistance to β-lactamase antibiotics, with penicillin or amoxicillin being the antibiotic of choice [30]. New recommendation strategies, first published in 2018 by NICE, propose a more conservative approach to antibiotic treatment of streptococcal pharyngitis if symptoms are light, last between 3 and 5 days, and the patient appears clinically well [46]. In that case, the treatment can be limited to self-care symptom management such as nonsteroidal anti-inflammatory drugs. Data on antibiotic resistance before and after the COVID-19 pandemic are depicted in Table 2. Schöbi et al. reported that the outbreak of GAS infection in 2022–2023 did not lead to an uprise in regards to antibiotic management in outpatient clinics in Switzerland [31]. During the recent GAS outbreak, beta-lactam antibiotic remained the antibiotic of choice, although the duration of use, the co-administration of other antibiotics, and the change in antibiotic schema were not homogenous among the studies [26,41]. Ramírez de Arellano et al., in a retrospective study of iGAS infection in children conducted in Spain in 2022–2023, concluded that all GAS isolates were susceptible to penicillin, while the resistance rate to tetracycline, erythromycin, and clindamycin was 3.8%, 4.6%, and 3.8% accordingly [47]. Maldonado-Barrueco et al. [14] reported that all iGAS cases were susceptible to beta-lactams, macrolides, clindamycin, and fluoroquinolones, whereas Li et al. [45] noted that GAS isolated appeared resistant against erythromycin (94.74%), followed by clindamycin (92.98%), and tetracycline (87.72%). Nack et al. [41] observed a resistance rate regarding GAS to clindamycin and erythromycin at 14.8% and 18.5%, respectively. Singer et al. reported no alteration in antibiotic resistance in iGAS cases in Germany during their study period 2017–2023 [23]. Barnes et al. [38] detected no changes in predicted antibiotic susceptibility in 34 iGAS cases observed in Colorado, USA, from October to December 2022. Data from Canada by Golden et al. [44] report a low antibiotic resistance in iGAS cases 2021–2022, with resistance to clindamycin remaining steady during the study period (around 4%) and no resistance to penicillin or vancomycin noted. A slightly increasing resistance to erythromycin was observed from 9.8% in 2018 to 14.1% in 2022. Nixon et al., in a retrospective study conducted in Australia between 2012 and 2023, noted that clindamycin and erythromycin resistance rates peaked in 2021 at 6.0% and 12.2%, respectively, and then returned to near baseline at 1–2% in 2023 [48].

## 4. Molecular Analysis

Data on molecular epidemiology are shown in Table 3. Molecular epidemiology research during the pandemic years is conducted by Ikebe et al. [11] regarding STSS cases and GAS types from 2019 to 2021 in Japan. They noted a decrease in the incidence of STSS induced by a change in the strain type. *Emm1* was dominant before the pandemic, while a decrease in this specific type was observed during quarantine. On the other hand, the *emm89* type remained in constant epidemiology during those years. During the same time period (2020–2021), Li et al. [45] studied GAS isolates from throat swabs and skin infection in China, concluding that *emm12* and *emm1* were the major types expressing smeZ, ssa, speG, speC, and smeZ, ssa, and speC, respectively. GAS skin infections were downregulated during the pandemic, and no correlation could be found between *emm* type, superantigen expression, and disease display.

After the implementation of IMS strategies and the subsequent rise in the GAS infection rate, many studies focused on the molecular epidemiology of GAS infection during that time frame. Data from the UK from January 2022 to March 2023 regarding the genome of iGAS cases by Vieira et al. [49] showed an M1UK lineage spike, isolated in 95.7% of cases, with three new clades identified during this uprise, while Lees et al. [26] also noted a predominance of *emm1* in pneumonia cases in the UK in the same time. Similarly, Holdstock et al. [27] also presented *emm1* as the main type causing GAS-related pleural empyema in children in the UK, 2022. Finally, Guy et al. [42] reported a rise in the incidence of iGAS incidences in children in England in 2022, with 14 deaths occurring from week 37 to 48 in 2022. A total of 25.8% of the pediatric iGAS cases reported a preceding viral infection, most frequently RSV or human metapneumovirus. In the pediatric population, *emm1* was the leading GAS type, followed by *emm12* and *emm4*. Of the fourteen deaths reported above, nine were caused by *emm1* type, the majority of whom identified as M1UK lineage, with speA expression. Alcolea-Medina et al. [50] conducted an *emm* typing analysis of GAS cases during the outbreak in London in December 2022, where 10.8 cases per day were diagnosed. A total of 76% were pediatric cases, with 10% being invasive, usually presenting as lower respiratory tract infections, including two deaths. *Emm12* was the dominant type (59%), followed by *emm1*, with 78% categorized as M1UK lineage. Most GAS that caused invasive disease expressed superantigen genes speA (70%) and speJ (80%). In regards to non-invasive GAS cases, 73% of them were pharyngitis. Maldonado-Barrueco et al. [14] assessed the molecular analysis of GAS responsible for bloodstream infections in Spain during the GAS outbreak alert in the UK (December 2022). *Emm1* was the main type (42.9%), with 75% of the isolates categorized as the M1UK clone, and *emm12* (14.3%) followed in frequency. Those data appeared quite similar to the molecular data available in the UK during the same period [49]. As far as superantigen genes, speG was dominant in frequency (85.7%), but speA and speJ were present in the M1UK clone. Ramírez de Arellano et al., in a retrospective study of iGAS infection in children conducted in Spain in 2022–2023, observed that *emm12* was the dominant type in iGAS infection isolated in children in 2022, while *emm1* was the main type in 2023. While the M1UK lineage was spread in 2023, it did not exceed the M1global lineage [47]. Similar data is reported by Wolters et al. [51], with the outbreak of *emm1*, M1UK clone in Germany in regards to iGAS infection in 2023, and by Peetermans et al. [5] in Belgium, with the supremacy of *emm1* strains (73%), 83% of them categorized as M1UK clone. Likewise, iGAS infection in Iceland in the same period was attributed to the M1UK lineage [52], with a similar pattern also detected by Gouveia et al. [53] in Portugal. The rise of pediatric iGAS infection in 2022–2023 in Portugal was mainly expressed as pneumonia with or without empyema and sepsis, followed frequently by viral infection, with a mortality rate of 5.1%. MI1UK lineage was the dominant find, followed by *emm12*. PICU admission was correlated with *emm1* isolation. In a multicenter, nationwide cohort study that took place in 2022–2023 in Denmark, Nygaard et al. [43] noted an increase in pediatric iGAS infection when compared with data from 2016 to 2019 (pre-pandemic surveillance). The most common iGAS manifestation was soft tissue infection and complicated pneumonia, presenting a 4-fold increase, but no increase in the severity of iGAS was noted. The authors also detected a rise in upper respiratory tract infections in children in 2022–2023. In contrast with the data reported from the UK, where emm1 was the leading cause of infection, the main GAS type was *emm12*, but there was no association between *emm* type and severity of the disease. Another study, also reporting data for the surveillance of iGAS from Denmark by Johannesen et al. [52], reported a threefold upregulation of iGAS infection mainly affecting children younger than 5 years old, with a peak in January 2023, following the downregulation of the pandemic period. Unlike the data reported by Nygaard et al. [43], the authors stated a new lineage (M1DK) was first detected in August 2022, derived from the M1 clone, responsible for 30% of iGAS isolates in 2023 all over Denmark. M1DK is not characterized by any mutations detected in the M1UK lineage but possesses a bacteriophage expressing the exotoxin speC. It was not correlated with a higher mortality rate or need for ICU admission. Valcarcel Salamanca et al. [33] described the epidemiological changes in types of GAS in iGAS infection in Norway. During the pre-pandemic years (2019–2020), the most frequent type was *emm1*, followed by emm28, with a shift in dominance during the COVID-19 pandemic, with *emm89* being the dominant type. After the pandemic years, emm1, followed by *emm12*, made a comeback. Mangioni et al. [16] studied GAS types responsible for invasive or noninvasive infection in Milan, Italy, in the years 2022–2023. The majority of GAS cases included upper respiratory tract infection, followed by necrotizing fasciitis. *Emm1* and *emm12* were the leading types, with *emm12* expressing speH and speI mainly found in children and no-iGAS infections. iGAS cases were mainly attributed to the *emm1* strain. Van Kempen et al. [34] isolated *emm12* in 38% and *emm1* in 25% of iGAS cases in the Netherlands in 2022, concluding that the upsurge of cases was not attributed to a single type. Rümke et al. [1] studied the molecular epidemiology of GAS-causing invasive and non-invasive disease in the years 2009–2023 in the Netherlands. An upsurge of *emm1* type, especially M1UK lineage, was noted when comparing data from the first trimester of 2022 and 2023, coinciding with a rise in iGAS infection. *Emm1* was isolated mainly from invasive disease cases, while their incidence in carriers remained stable. M1UK lineage had a high rate among invasive cases, while M1global characterized mainly asymptomatic carriers. An expansion of four new clades of M1UK was also observed. Van der Putten et al. [54], in a study that took place also in the Netherlands in 2022, described an increase in iGAS cases in the pediatric population, with a high occurrence of *emm4* type, in contrast with the findings of van Kempen et al. [34]. A new lineage (M4NL22) of *emm4* was responsible for 85% of iGAS cases in 2022, exhibiting many genetic changes compared to other *emm4* strains. Data from the USA, and particularly Eastern North Carolina in 2022–2023, as noted by Huang et al. [55], report changes in GAS typing before and after the pandemic, with an unusual sublineage ST28/*emm1*, while Barnes et al. [38] underlined the high frequency of *emm1* type in iGAS infection in Minnesota in 2022. Ho et al. [40], on the other hand, showed an increase in M12 strains in 2022 in Colorado, compared to pre-pandemic years. In the same manner, Nack et al. [41] reported *emm1* and *emm12* as the most common types of GAS responsible for invasive GAS infection in children younger than 1 year of age in the USA. Data from Canada by Golden et al. [44] also report a downregulation in iGAS cases in 2021 during quarantine and a subsequent rise in iGAS cases in the last trimester of 2022, mostly attributed to pediatric cases in children < 15 years old. The dominant type was *emm49* during 2021–2022, although a quick rise in *emm1*, 49% of which was M1UK, and *emm12* was noted at the end of 2022. The dominance of emm1 type in iGAS cases was underlined by Abo et al. [36] in a study conducted in Australia in 2022.

**Table 3:** Studies on the molecular epidemiology of GAS infection before and after the COVID-19 pandemic.

First Author, Publication Year	Country	Study Type	Time Period	Study Design	Study Population	Results
Maldonado-Barrueco et al. [14], 2024	Spain	cohort	2017–2023	retrospective	35 patients (adults and children	Genomic epidemiology in 2023 in Spain is similar to the reported data from the UK outbreak alert in the same period. *Emm1* was the main type (42.9%) detected.
Ramírez de Arellano et al. [47], 2024	Spain	Observational	2022–2023	Retrospective	130 isolates of *S. pyogenes*	*Emm12* was the dominant type of iGAS infection isolated in children in 2022, while *emm1* was the main type in 2023.
Vieira et al. [49], 2024	UK	cohort	2022–2023	retrospective	1092 laboratory samples	The upsurge in invasive infections was associated with a significant increase in *emm1 S. pyogenes*, the vast majority (95.7%) of which belonged to the emergent M1UK lineage or its derivatives.
Alcolea-Medina et al. [50], 2023	UK	observational	2022	prospective	56 isolates	*Emm12* and *emm1* types predominate in the ongoing outbreak in 2022 in the UK, which mainly affects children.
Guy et al. [42], 2023	UK	observational	2022	retrospective	772 patients (adults and children)	In the pediatric population, *emm1* was the leading GAS type, followed by *emm12* and *emm4*.
Holdstock et al. [27], 2023	UK	case series	2022	retrospective	16 children < 16 years old	*Emm1* as the main type causing GAS-related pleural empyema in children in the UK, 2022.
Nygaard et al. [43], 2024	Denmark	cohort	2016–2017, 2021–2023	retrospective	174 children < 18 years old	In Denmark, the incidence of pediatric iGAS increased in 2022–23. The main GAS type was *emm12.*
Johannesen et al. [52], 2023	Denmark	cohort	2018–2023	retrospective	1265 laboratory samples	The recent surge in Denmark in iGAS cases coincided with the rise of a novel lineage (M1DK).
Mangioni et al. [16], 2024	Italy	cohort	2022–2023	retrospective	179 patients (19 adults and 9 children)	*Emm1* and *emm12* were the leading types of GAS infections, with *emm12* expressing speH and speI mainly found in children and non-iGAS infections. iGAS cases were mainly attributed to the *emm1* strain.
Peetermans et al. [5], 2024	Belgium	cohort	2022–2023	retrospective	86 patients (56 adults and 30 children)	A high number of admissions to Belgian tertiary critical care units of patients with severe invasive *S. pyogenes* infections associated with the introduction of the M1UK lineage.
Wolters et al. [51], 2024	Germany	cohort	2022–2023	retrospective	47 patients	Hypertoxigenic Streptococcus pyogenes *emm1* lineage M1UK is present in Germany and might constitute a driving force in the observed surge of GAS infections.
Gouveia et al. [53], 2023	Portugal	cohort	2022–2023	retrospective	89 children < 18 years old	Invasive group A Streptococcus infections in Portugal (*n* = 89) were higher than in pre-COVID-19 seasons, dominated by the M1UK sublineage.
Valcarcel Salamanca et al. [33], 2024	Norway	cohort	2015–2024	retrospective	2129 patients	During the pre-pandemic years (2019–2020), the most frequent type of iGAS infection was *emm1*, followed by emm28, with a shift in dominance during the COVID-19 pandemic, with *emm89* being the dominant type. After the pandemic years, emm1 followed by *emm12* made a comeback.
van Kempen et al. [34], 2022	Netherlands	observational	2018–2019, 2021–2022	retrospective	117 children < 18 years old	*Emm12* was found in 38% and *emm1* in 25% of iGAS cases in the Netherlands in 2022.
van der Putten et al. [54], 2023	Netherlands	cohort	2009–2019, 2022	retrospective	66 isolates	Invasive group A streptococcal (iGAS) disease cases increased in the first half of 2022 in the Netherlands, with a remarkably high proportion of *emm4* isolates.
Rümke et al. [1], 2024	Netherlands	cohort	2009–2023	retrospective	3049 children and adults	High iGAS incidence between March 2022 and March 2023 in the Netherlands coincided with a marked expansion of *emm1* among iGAS isolates.
Barnes et al. [38], 2023	USA	observational	2016–2019, 2020–2021, 2022	retrospective	49 children < 18 years old	High frequency of *emm1* type in iGAS infection in Minnesota in 2022.
Huang et al. [55], 2024	USA	observational	2015–2021, 2022–2023	retrospective	13,159 isolates	Epidemiological changes before and during the COVID-19 pandemic in Easter North Carolina detected a unique sub-lineage in ENC among the most common invasive GAS strain, ST28/*emm1*.
Ho et al. [40], 2023	USA	observational	2022–2023	prospective	96 patients < 21 years old	Outbreak case numbers of iGAS were almost triple the pre-pandemic baseline, with an increase in M12 strains in 2022 in Colorado.
Nack et al. [41], 2024	USA	observational	2012–2022	retrospective	32 infants < 1 year old	*Emm1* and *emm12* are the most common types of GAS responsible for invasive GAS infection in children younger than 1 year of age in the USA.
Golden et al. [44], 2024	Canada	Observational	2021–2022	Retrospective	4809 patients	The dominant type in iGAS infection was *emm49* during 2021–2022, although a quick rise in *emm1*, 49% of which was M1UK, and *emm12* was noted at the end of 2022.
Ikebe et al. [11], 2024	Japan	observational	2019–2022	retrospective	526 patients (adult and children)	*Emm1* was the dominant type of GAS bloodstream infection before the pandemic, while a decrease in this specific type was observed during quarantine. *Emm89* type remained of constant epidemiology during those years.
Li et al. [45], 2023	China	cohort	2020–2021	retrospective	114 children	Under the COVID-19 pandemic, GAS infection cutaneous diseases decreased dramatically. There was a correlation between *emm*, the superantigen gene, and disease manifestations.
Abo et al. [36], 2023	Australia	observational	2018–2022	retrospective	280 children < 18 years of age	The dominance of emm1 type in iGAS cases was noted in Australia in 2022.

## 5. Discussion

Streptococcus pyogenes is a pathogen causing a great spectrum of disorders, from asymptomatic colonization of the oropharynx to invasive disease. Although streptococcal pharyngitis is a common pediatric disease occurring in winter and springtime, responding in the majority of cases at per os antibiotic administration, invasive disease portrays a challenge, not only diagnostically but also in regards to management and treatment, with a high rate of PICU admission, morbidity, and mortality [1].

In December of 2022, the WHO brought the upsurge of invasive GAS infection to attention, followed by the same remark by the CDC later that year. This alert instigated epidemiological research in regard to GAS infection, not only retrospectively but also prospectively, in an effort to imprint current trends, mortality, and molecular epidemiology in many countries all around the globe [5].

During the COVID-19 pandemic, a downregulation in GAS infection in Europe and the USA was noted [37], affecting invasive and non-invasive disease. Streptococcal pharyngitis was decreased by 81.6% in the USA during the pandemic [13], an observation coinciding with epidemiological data from many European countries, such as France [19], Italy [6], Spain [4], and the UK [24]. A similar note was observed about invasive infection, with a reduction in GAS bloodstream and soft tissue infection, pneumonia, and STSS cases [18,37]. At the end of 2022, when IMS were overridden, an increase in GAS infection was underlined. As far as mild infection is concerned, for example, scarlet fever [4] and strep throat [19,30,33,50], a rise overcoming pre-pandemic levels was remarked, with cases following no seasonal peak [6,24,38]. In Italy, 20.3% of children visited the family doctor for GAS-related infection such as tonsillopharyngitis at the beginning of 2023 [30], while in France a rise of +23.8% per month in tonsillitis cases during 2022 was noted [19]. This uprise continued in countries such as Norway during the year 2023, with a peak in pharyngitis cases in December 2023, as reported [33].

As to invasive GAS infection, a proliferation in cases after December 2022 was underlined by many studies in Europe [5,25,28], the USA [38,39,41], and China [45], a trend that continues in some countries until January 2024 [33]. IGAS cases manifest usually as complicated pneumonia with empyema or parapneumonic effusion, followed by soft tissue infection, bloodstream infection with no focus, and STSS. Complicated pneumonia cases usually occur with no seasonal pattern in children younger than 5 years old, with rapid clinical deterioration, leading to longer hospital stays, high rates of PICU admission, surgical management, and a high mortality rate, as portrayed by data from Poland [28], the UK [26], Belgium [5], Spain [14], Germany [21], the Netherlands [35], Australia [36], and the USA [41]. Specifically, data from the UK report a high death rate of 25% [25]. On the contrary, data from Switzerland [31], Germany [22], and the USA [38] observed no longer hospitalization or PICU admission in children with GAS-related pneumonia and other iGAS infections. In general, the majority of studies agree that iGAS cases coincided with or followed viral infection, especially RSV, Influenza A/B, Varicella zoster, and human metapneumovirus [21,26,27,34]. In 2022 and 2023, a surge in viral outbreaks was noted, especially concerning RSV and Influenza A/B, designating no seasonal pattern, with Influenza B continuing until the late spring of 2023 [31].

Regarding the molecular epidemiology of GAS, the *emm1* strain was reduced during the pandemic years in Japan [11], while *emm89* remained at the same incidence levels, coinciding with the reduction in invasive GAS infection during this time. An equivalent observation is noted also from epidemiological data from Norway [33]. Simultaneously, a study in China [45] showed that *emm1* and *emm12* remained the dominant types during quarantine. During the alert period of invasive GAS infection in Europe, the *emm1* type was the primary type isolated in the plethora of iGAS cases, with *emm12* following in frequency. The supremacy of the *emm1* type is apparent in data from Spain [14], Belgium [5], Germany [51], Portugal [53], Norway [33], the Netherlands [1], Italy [16], the UK [49], Australia [36], and Minnesota, USA [38], while data from other studies from Denmark [43], the Netherlands [34], and Colorado, USA [40] concluded that *emm12* was the mainly isolated type. *Emm* typing could not be correlated with the severity of disease in the majority of cases [43], although a study linked the emm1 strain with invasive disease and *emm12* with non-invasive disease [16]. An emerging lineage of *emm1* with high virulence, M1UK, surfaced in the post-pandemic period, expressing the exotoxin speA [50]. This lineage was correlated with invasive disease, and while it has existed since 2019, it reached high levels of transmission and dominance after the pandemic in several European countries, such as the UK [49], Portugal [53], Germany [51], Belgium [5], France [19], Spain [14], Iceland [52], and the Netherlands [1]. Specifically, in the UK, where it was first isolated, M1UK was linked with a high rate of mortality in the pediatric population [42]. Besides M1UK, another lineage was recognized in Denmark, M1DK, first discovered in August 2022. This lineage emerged as the dominant type responsible for iGAS cases in Denmark in 2022, expressing exotoxin speC [52]. Concurrently, a new lineage (M4NL22) of *emm4* emerged in the Netherlands in 2022, responsible for the majority of cases [54].

Many theories about this unusual surge of GAS cases occurring after the pandemic period have risen. One possible explanation is that the implementation of infection mitigation strategies, such as hand washing, social distancing, online education programs, and avoidance of overcrowding, led to decreased exposure to GAS. As a result, young children lacked immunity to GAS, as they did not achieve adequate immunity levels against GAS during the pandemic, leading to a plethora of children being vulnerable to the disease. Specifically, young children, who had never been exposed to the pathogen before the pandemic and were first exposed after the implementation of IMS strategies, were more vulnerable to GAS infection. Nevertheless, even older children and adults who had been exposed to the pathogen before were not able to boost their immunity levels during the pandemic, leading to a decrease in herd immunity [13,56,57]. Furthermore, the rise of viral infection waves such as RSV, Influenza, Varicella zoster, and human metapneumovirus after the COVID-19 pandemic is a plausible cause of the rise of GAS cases, increasing the risk for invasive disease, a correlation depicted by many studies [4,20,23,25,28]. Finally, emerging *emm* types, such as *emm1* and *emm12*, but most importantly, the highly virulent M1UK [42,53] and M1DK lineage [52], are also proposed as possible explanations for the high rates of invasive disease. Nevertheless, the diversity of *emm* types isolated in different countries that experience the same uprise in GAS infection suggests that a single variant is not at fault [36].

As GAS is the cause of invasive disease with a high mortality rate, healthcare professionals need to be alert for the wide clinical manifestation of the disease spectrum, leading to early identification, prompt treatment, and superior clinical outcome. A GAS vaccine targeting the M gene is a field of great research, and its priority has been highlighted by WHO ever since 2018 [43]. As cases of GAS infection affect low- and high-income countries, this effort demands global collaboration. Despite over a century of research, a commercially available GAS vaccine has yet to be developed due to various historical, scientific, and economic challenges. A major obstacle in vaccine development is that *S. pyogenes* antigens contain autoimmune epitopes, which can trigger acute rheumatic fever (ARA). This risk led to a 25-year ban on *S. pyogenes* vaccine trials in humans by the FDA, lifted only in 2005 [58]. Additionally, the complex epidemiology of *S. pyogenes*, including over 240 *emm* types and varying disease patterns, complicates vaccine research. The M protein is considered a key antigen in GAS vaccine development, alongside several non-M protein fragments. Recent advances in both preclinical and clinical settings suggest that M protein-based vaccines hold greater potential compared to non-M protein-based alternatives. Peptide-based subunit vaccines, which use minimal peptides derived from the M protein (such as J8, J14, and P145), are promising in inducing long-lasting immunity without triggering autoimmune responses or inflammatory reactions typically seen with traditional vaccines [59,60,61]. While the low immunogenicity of these subunit vaccines requires strong immunostimulators to elicit a robust adaptive immune response, their safety and efficacy have been demonstrated in early preclinical and clinical studies. Although substantial progress has been made in various animal models, clinical development faces significant challenges, including a lack of defined immune correlates of protection in humans, limited animal models, diverse GAS strains, and concerns over potential autoimmune complications [62,63].

## 6. Conclusions

The COVID-19 pandemic and the implementation of IMS strategies led to a downregulation of GAS infection, a circumstance that was altered once they were discontinued. A global surge in GAS infection (invasive and non-invasive) was noted, especially concerning the pediatric population. Children younger than 5 years old were most affected. Complicated pneumonia was the leading clinical manifestation, causing many deaths worldwide. M1UK was recognized as the dominant lineage in Europe and was correlated with invasive disease. The growing incidence of invasive GAS infections in children highlights the urgent need for ongoing surveillance of *emm* types and superantigens, as well as the establishment of clear diagnostic and treatment guidelines. Early initiation of supportive care and broad-spectrum antibiotics, including β-lactams and clindamycin for their anti-toxin effects, is crucial. Enhancing awareness of the disease’s clinical presentation, risk factors, and the latest diagnostic and therapeutic advances is essential to improving outcomes in pediatric patients.

## Figures and Tables

**Table 2:** Studies on antibiotic resistance of GAS infection before and after the COVID-19 pandemic.

First Author, Publication Year	Country	Study Type	Time Period	Study Design	Study Population	Results
Maldonado-Barrueco et al. [14], 2024	Spain	cohort	2017–2023	retrospective	35 patients (adults and children	All iGAS cases were susceptible to beta-lactams, macrolides, clindamycin, and fluoroquinolones
Ramírez de Arellano et al. [47], 2024	Spain	Observational	2022–2023	Retrospective	130 isolates of *S. pyogenes*	All GAS isolates were susceptible to penicillin, while the resistance rate to tetracycline, erythromycin, and clindamycin was 3.8%, 4.6%, and 3.8%, respectively.
Li et al. [45], 2023	China	cohort	2020–2021	retrospective	114 children	GAS isolated appeared resistant against erythromycin (94.74%), followed by clindamycin (92.98%), and tetracycline (87.72%)
Schöbi et al. [31], 2024	Switzerland	observational	2013–2023	retrospective	284 children < 16 years old	Outbreak of GAS infection in 2022–2023 did not lead to an uprise in regards to antibiotic management, with beta-lactam antibiotic being the antibiotic of choice
Singer et al. [23], 2024	Germany	observational	2017–2019, 2022–2023	retrospective	4885 invasive isolates	No alteration in antibiotic resistance in iGAS cases in Germany during 2017–2023
Barnes et al. [38], 2023	USA	observational	2016–2019, 2020–2021, 2022	retrospective	49 children < 18 years old	No changes in predicted antibiotic susceptibility in 34 iGAS cases observed in Colorado, USA
Nack et al. [41], 2024	USA	observational	2012–2022	retrospective	32 infants < 1 year old	Resistance rate regarding GAS to clindamycin and erythromycin is 14.8% and 18.5%, respectively.
Golden et al. [44], 2024	Canada	Observational	2021–2022	Retrospective	4809 patients	Low antibiotic resistance in iGAS cases
Nixon et al. [48], 2024	Australia	Observational	2012–2023	Retrospective	33,519 GAS isolates	Clindamycin and erythromycin resistance rates peaked in 2021, at 6.0% and 12.2%, respectively, and then returned to near baseline at 1–2% in 2023.

## Data Availability

Data are contained within the article.
